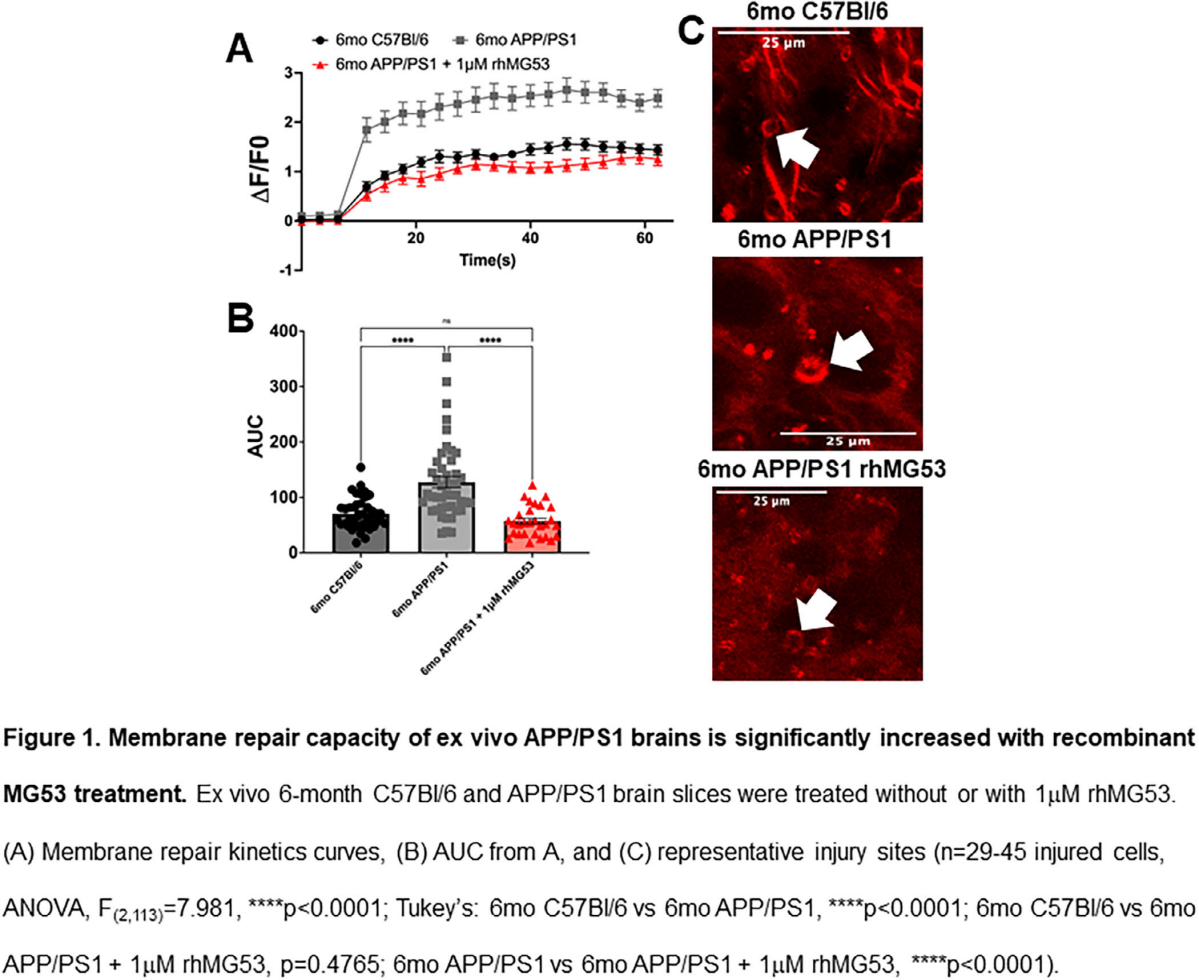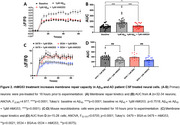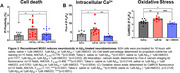# Recombinant TRIM72/MG53 protein enhances plasma membrane repair and reduces neurotoxicity in models of Alzheimer’s Disease

**DOI:** 10.1002/alz70859_096157

**Published:** 2025-12-25

**Authors:** Hannah R Bulgart, Miguel A Lopez Perez, Gianni N Giarrano, Loren Wold, Douglas W. Scharre, Noah Weisleder

**Affiliations:** ^1^ University of Kentucky, Lexington, KY USA; ^2^ The Ohio State University, Columbus, OH USA; ^3^ Ohio State University Wexner Medical Center, Columbus, OH USA

## Abstract

**Background:**

Amyloid beta (Aβ) is one of the earliest hallmarks in Alzheimer’s Disease (AD) that has been shown to localize with the plasma membrane, decrease membrane integrity, elevate intracellular calcium concentrations, increase oxidative stress and directly induce membrane damage. Compensation for such damage to the plasma membrane requires a robust repair mechanism to avoid cell death. Our recent published studies demonstrated a plasma membrane repair defect in neurons within the APP/PS1 mouse brain, which overexpress Aβ, and with the application of AD patient cerebrospinal fluid (CSF) samples to primary neurons. *Here we tested if enhancing membrane repair with recombinant TRIM72/MG53 (rhMG53) protein could reduce neurotoxicity and cell death*.

**Method:**

To analyze the effect of rhMG53 on membrane repair, we exposed 6‐month C57Bl/6 and APP/PS1 mouse brain slices to 1µM rhMG53 and conducted the laser damage assay where a two‐photon laser is used to ablate a portion of the plasma membrane in the presence of FM4‐64, and dye entry was used as a proxy for repair capacity. We repeated this assay on primary neurons treated with rhMG53 and recombinant Aβ or AD patient CSF. Lastly, we measured cell death by propidium iodide staining, intracellular calcium by Fluo‐4 staining, and oxidative stress by CellROX staining in the presence and absence of rhMG53.

**Result:**

Our results demonstrated a significant increase in membrane repair capacity in APP/PS1 brain slices treated with rhMG53 compared to untreated slices, and no significant difference between C57Bl/6 control slices indicating a rescue to baseline repair kinetics (Figure 1). Furthermore, we observed a significant increase in membrane repair capacity when primary neurons were treated with rhMG53 in conjunction with Aβ or AD CSF (Figure 2). Lastly, we observed a significant decrease in cell death and neurotoxicity markers when treated with Aβ and rhMG53 compared to Aβ treated cells (Figure 3).

**Conclusion:**

These results indicate using rhMG53 to enhance plasma membrane repair capacity can compensate for downstream neurotoxicity in AD neurons induced by Aβ. Our future studies will focus on treating AD mouse models with rhMG53 to determine if the protein can enhance spatial learning and memory and other aspects of AD pathology.